# Born global: the influence of international orientation on export performance

**DOI:** 10.1016/j.heliyon.2019.e02688

**Published:** 2019-11-14

**Authors:** Diana Escandon-Barbosa, Josep Rialp-Criado, Sascha Fuerst, Augusto Rodriguez-Orejuela, Geovanny Castro-Aristizabal

**Affiliations:** a Pontificia Universidad Javeriana, Colombia; b Autonoma of Barcelona University, Spain; c Eafit University, Colombia; d Universidad Del Valle, Colombia

**Keywords:** Business, Market dynamism, Export performance, Innovative capacity, International orientation

## Abstract

New international enterprises that are referred to as Born Globals have become the subject of research due to the success of their global operations, despite their early internationalization and limited resources. Given the importance of analyzing the characteristics that contribute to the success of Born Globals, our study examines the influence of international orientation on export performance. Additionally, we consider internal and external drivers for early and accelerated internationalization such as the Born Global's innovative capacity, the dynamism of the market and the favorability of the environment. By estimating a structural equation model, the results show that international orientation is a strong driver of the export performance of Born Globals. However, this relationship is moderated by innovative capacity and the dynamism and favorability of the environment, thus achieving a greater effect on export performance when international orientation is complemented by these variables.

## 1 Introduction

Born Globals (BGs) are defined as “entrepreneurial start-ups that, from or near their founding, seek to derive a substantial proportion of their revenue from the sale of products in international markets.” (Knight and Cavusgil, 2004, p. 124). This type of firm contrasts companies that follow a gradual path of internationalization often related to the so-called internationalization process theory (Johanson and Vahlne, 1977). The internationalization process theory describes an approach to international markets whereby firms gradually increase their international involvement by entering foreign markets initially through independent representatives before subsequently establishing their own sales and production facilities. Firms reduce their perceived uncertainty about operating abroad mainly through a process of experiential learning entering nearby and culturally familiar markets first before conquering geographically and culturally distant markets. This gradual path of internationalization contrasts the early international market entry and rapid pace of internationalization of BGs (Paul and Rosado-Serrano, 2019). Therefore, the internationalization process theory is not able to explain the accelerated internationalization of BGs (Freeman et al., 2010; Lopez et al., 2009; McDougall et al., 1994).

It is therefore important to ask, what are the characteristic of BGs that distinguish them from firms that follow the gradual and incremental path of internationalization? These characteristics relate to internal and external factors or drivers for early and accelerated internationalization (Hagen and Zucchella, 2014; Madsen and Servais, 1997; Rialp et al., 2005). One of the internal key drivers relates to the founder's experience and background (Loane et al., 2007; Madsen and Servais, 1997; McDougall et al., 1994). The founder's global vision at the firm's inception and his or her commitment towards international markets is an important predictor for the early and rapid internationalization of BGs. The external factors that contribute to the born global phenomenon are associated, one the one hand, with the globalization of production and markets, and, on the other hand, with technological advances in information and communication technologies that enable innovative business models targeting international or global markets near inception of the firm (Knight and Cavusgil, 2004; Knight and Liesch, 2016; Mathews and Zander, 2007). Hence, BGs often target niche markets with innovative products to globally dispersed clients using internet-enabled distribution methods. BGs are thereby active in technology and knowledge-intensive industries compared to gradual internationalizer that often operate in traditional manufacturing industries or labor-intensive or small-scale industries (Paul and Rosado-Serrano, 2019).

The internal capabilities and competences are the most important aspects for small firms to succeed in international markets (Knight and Cavusgil, 2004; Wu et al., 2007; Zahra et al., 2000). This is due to their lack of tangible resources compared to their more resource-rich, larger counterparts. Considering the salience of the firm's vision and commitment towards international market as a key driver for BGs, we choose the construct of international orientation (Knight and Kim, 2009; Sorensen and Madsen, 2012) in order to research its relationship to export performance. Managements' international orientation has been researched in many studies focusing on export behavior and several literature reviews point out that this strategic orientation is positively related to export success (Aspelund et al., 2007; Knight and Kim, 2009; Leonidou and Katsikeas, 1996; Sorensen and Madsen, 2012). However, the construct has not been applied in the context of BGs. This is surprising since the international orientation of the founders of the BG is considered an important characteristic that differentiates this type of firm from gradual internationalizers.

Considering both the internal push and the external pull effects of internationalization (Mathews and Zander, 2007), we are also interested in exploring the moderating effects on the relationship between international orientation and export performance. As previously mentioned, BGs often operate in technology and knowledge-intensive industries compared to gradual internationalizer. Innovation becomes a tenet in these markets in order to achieve sustainable, superior performance (Efrat and Shoham, 2012; Knight and Cavusgil, 2004). We therefore consider the BG's innovative capacity (Luo et al., 2005) as a moderator between the firm's international orientation and its export performance. External pull effects within the environment of the BG are also able to moderate this relationship. We therefore take into account the market dynamism (Jaworski and Kohli, 1993) and the favorability of the business environment (Sutcliffe and Huber, 1998) as moderators. Considering these moderators, we also answer calls for more research on the effects of variables related to the environment and context of BGs (Knight and Liesch, 2016).

Altogether, in this study we are interested in exploring how BGs achieve superior international performance from a capability perspective. We thereby focus on international orientation and its relationship to export performance considering the impact of internal and external drivers on this relationship. Doing so, we answer calls for more research that relates to: 1) Providing explanations of how BGs are able to achieve precocious internationalization and superior international performance (Knight and Liesch, 2016); 2) Analyzing the effects of variables related to the environment and context of BGs (Knight and Liesch, 2016); 3) Contribute research in the context of a developing country (i.e. Colombia) (Baier-Fuentes et al., 2019; Oyna and Alon, 2018; Paul and Rosado-Serano, 2019).

In the following section we derive the conceptual model based on the review of literature. This is followed by a description of the methodology in section 3. In section 4 we present the results of the main effects and the moderating effects following Aiken and West (1991). For the discussion in section 5, we first summarize the results before elaborating on the theoretical contributions of our findings. Subsequently, we present the managerial implications. We conclude with an acknowledgment of the limitations of our study and the directions for future research.

## 2 Hypothesis

Exporting remains an important strategic option for firms to internationalize and is the most frequently used entry method due to its cost-effectiveness and high flexibility as compared to other foreign market entry modes such as foreign direct investments (Sousa et al., 2008). Exporting also serves as the major foreign entry mode for firms from developing countries (Singh, 2009). Due to the importance of exporting for many firms’ growth and survival, there is an increasing interest in understanding the drivers for export performance (Chen et al., 2016).

Studies on the export performance in BGs focus on capabilities for entering and operating in international markets that include entrepreneurial skills to exploit resources and capacities, acquiring new technologies and entering new markets, and adapting such resources and capabilities to take advantage of market opportunities (Gerschewski et al., 2015; Holmqvist, 2004; Jin et al., 2018; O'Reilly and Tushman, 2008; Teece, 2007).

Taking into consideration that we are interested in understanding how BGs achieve superior international performance, we are adopting a capability perspective. Earlier research shows that internal capabilities are able to offset the resource-disadvantage of smaller firms for achieving early and rapid internationalization particularly regarding tangible resources (Autio et al., 2000; McDougall et al., 1994; Zahra et al., 2000). This perspective is rooted in the resource-based view of the firm (Barney, 1991; Wernerfelt, 1984). A reviewer of this article points to the ownership advantage of Dunnings's eclectic paradigm (Dunning, 1980) as preceeding the resource-based view of the firm by Wernerfelt. We agree with it but also acknowledge that the ownership advantage as the competitive advantage of the firm rather explains the motivation for an enterprise to engage or increase its existing foreign direct investment, whereas, we are rather interested in explaining the expansion of firms through the export mode of foreign market entry.

According to the resource-based view of the firm (Barney, 1991; Wernerfelt, 1984), in order for a firm to create a competitive advantage it needs resources that are valuable, rare, inimitable, and non-substitutable. In our research we focus on intangible resources due to their difficulty to imitate by competitors and due to their relevance for achieving sustained competitive advantage and, therefore, superior performance (Hall, 1993).

Among the strategies that are adopted by BGs in order to achieve early and rapid internationalization, an international orientation is a critical factor as it represents the mentality and the attitude of the entrepreneur and decision-maker for international expansion (Dahms, 2015; Jin et al., 2018; Knight, 2000; Reuber and Fischer, 2002). An international orientation is considered to be a strategic orientation that is used by firms that seek to improve international performance (Sorensen and Madsen, 2012). Other strategic orientations include market focus (Ahimbisibwe et al., 2013; Cadogan et al., 2006; Kohli and Jaworski, 1990; Narver and Slater, 1990; Olimpia et al., 2006; Zhou et al., 2005), entrepreneurial focus (Strömberg and Bindala, 2012; Okpara, 2009; Patel & D'Souza, 2009; Strömberg and Bindala, 2012), learning focus (Andersson, 2004; Moen et al., 2004; Weerawardena et al., 2007; Wu, 2007), and innovation focus (Cadogan et al., 2003; Kirbach and Schmiedeberg, 2006; Wagner, 2001).

International orientation refers to entrepreneurs' attitudes and the assignment of resources to international activities (Sorensen and Madsen, 2012). International orientation has been studied in various empirical research on internationalization processes (Knight and Cavusgil, 2004; Knight and Kim, 2009; Madsen, 1989) and is considered to be a part of management's mentality. This is because an international orientation depends on seeing the world as one's market within which to create the motivation to negotiate with international clients (Sorensen and Madsen, 2012). Jantunen et al. (2008) argue that firms with an international orientation search for new methods of market entry that can allow them to expand and they are therefore willing to invest more resources to achieve this goal. Moreover, Heinonen et al. (2004) note that an international orientation allows firms to be differentiated according to their motivation for expansion in international markets.

Shoham (1998) brings together different conceptualizations of export performance, highlighting authors, such as Aaby and Slater (1989), Madsen (1987) and Shoham (1991), who in their definition include the effectiveness and efficiency of exports and the continued commitment of enterprises to export activities. Moreover, Cavusgil and Nevin (1980) note that the analysis of export performance should not only include the performance that is generated but also all of the decisions that are made regarding international activities. Cameron (1986) finds four aspects that should be considered in evaluating a firm's export performance: the international strategy that is used, sales performance, the impact of the internal activities of the firm, and the environment in which the firm operates. Subsequently, Zou et al. (1998) argue that export performance refers to the level of satisfaction with export operations as an indication of the international activity's success. Other studies define export performance as the results of a firm's activities in international markets (Cadogan et al., 2003). Leonidou et al. (2002) suggest the following export performance indicators: export intensity, growth of international sales, export profit level, volume of international sales, and market share. In addition to these indicators, Olimpia et al. (2006) opt to use subjective measures in relation to firm expectations.

In our study, we adopted the definition by Zou et al. (1998) to analyze export performance. We choose this definition because it focuses on the joint action that concerns the export product and the market, thus overcoming various difficulties in measuring export performance by integrating the three criteria that have been used in other studies: financial performance, strategic performance and satisfaction of the enterprise export activities. Although the purpose of this investigation does not involve the decomposition of these three criteria for the purposes of analysis, conceptualization and measurement scale, Zou et al. (1998) considers all three criteria of export performance for a company because successful performance is achieved through the analysis of sales growth without neglecting the strategic objectives of export companies. These objectives include the strategic position and the market share. Additionally, a firm's satisfaction with export results indicates a higher perceived success. Therefore, the firm's satisfaction with export results is able to reinforce its attitudes towards export and increases its propensity to expand its international operations (Zou et al., 1998).

Innovative capacity is considered to be a strategic element in firms as it allows the development of practices that are able to promote innovative activities among employees (Atuahene-Gima and Ko, 2001). Innovative capacity is considered an ability to produce a new process, product, or idea within an organization (Damanpour, 1991; Hult et al., 2004; Hurley and Hult, 1998) by involving different agents in the organization through a process of cultural change. In our study, innovative capacity is considered an internal driver that enhances the effects of international orientation on the export performance of BGs. This capacity is considered an intangible resource and key driver for generating a competitive advantage for the firm.

Related to the external drivers for firm performance, Shoham (1999) points to the need to assess changes in business conditions in environments where national and international markets interact. Therefore, a clearer understanding of the relationship between export performance and an international orientation can be obtained by analyzing factors that are related to the business environment. Two factors affect the quantity and complexity of the market information that is needed for managers with an international outlook: Market characteristics and industry characteristics (Lorhke et al., 1999). Markets change constantly, therefore, obtaining the required information for a firm that attempts to enter new international markets can be difficult due to an increased uncertainty (Johanson and Vahlne, 1977).

**Fig. 1:**
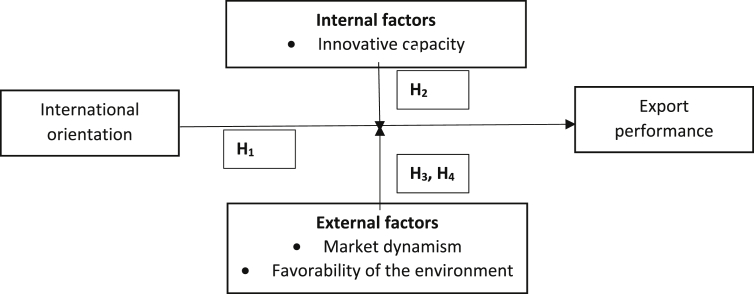
Conceptual model.

According to Sutcliffe and Huber (1998), the market dynamism is a condition of the environment that affects corporate behavior and is defined as the rate of change and instability of the environment in which the organization operates (Dess and Beard, 1984). This includes the actions of entrepreneurs, customers, suppliers, partners and the community in general that over time can affect the decision-making of an organization. This study adopts the concept of market dynamism that Jaworski and Kohli (1993) defined as market changes that are caused by customers and competitors. Therefore, given the existence of a dynamic international market, a firm should analyze the needs and preferences of its clients. Thus, firms operating in dynamic international environments tend to promote a high degree of international focus. Moreover, according to Sutcliffe and Huber (1998), the favorability of the environment also affects export performance, which represents the availability of the resources that exist in the environment that can be used by companies. This variable assesses the potential growth of organizations, given the greater availability of resources (provided by a favorable environment) and greater opportunities to access international markets with better results for the firm (Wan and Hoskisson, 2003). Based on the findings of previous studies that have found the business environment to be a moderating factor in export success and the different associated strategic foci (Zhou, 2006), we consider that the strategies that are adopted by firms to become international should factor into how such firms can adapt to their business environment. Therefore, the present study focuses on the moderating effect of the environmental attributes of market dynamism and the favorability of the business environment. Fig. 1 shows the conceptual model that has been developed in the present study.

### 2.1 International orientation and export performance

Previous research proposes a positive relationship between international orientation and export performance (Behyan et al., 2015; Jantunen et al., 2008; Knight and Kim, 2009; Sorensen and Madsen, 2012; Martin et al., 2018; Racela and Thoumrungroje, 2014), suggesting that the way that managers think reflects the international strategies that have been adopted by their firms. Furthermore, the international orientation of the firm can lead to the promotion of opportunity-seeking in foreign markets among employees.

Studies find that an international orientation provides the ability of a firm to enter international markets (Michel and Hambrick, 1992). Sorensen and Madsen (2012) conclude that an international orientation leads to a greater capacity to compile and interpret key information on international markets that can be used to make decisions to enter such markets. In the case of BGs, Knight (2000) notes that an international orientation allows these firms to develop strategic initiatives that are aimed at improving organizational performance. The author also argues that experience with internationalization and expansion to different markets allows a firm to learn more about foreign markets and therefore ensure a greater possibility of success. In other words, the rapid internationalization of BGs demonstrates that their employees know the environment in which they operate. Therefore, their operations in export markets become routines.

The international orientation of the founders, managers and employees of BGs allows them to acquire the abilities that are necessary to maintain their competitive advantage. This makes them more willing to assume risk when they expand into new markets and improves their export performance (Kuivalainen et al., 2007; Martin et al., 2018). Given the literature's emphasis on the importance of an international orientation, we hypothesize:

Hypothesis 1 An international orientation has a positive effect on the export performance of BGs.

### 2.2 Moderating effects

#### 2.2.1 International orientation and innovative capacity

The resource-based view proposes that the importance of internal resources is a key factor for achieving a competitive advantage. Barney (1991, 2001) discussed the impact of innovation capacity on export performance. At the company level, Teece (1996) argues that innovative companies have greater incentives to enter new international markets, thereby generating increased profitability.

Knight and Cavusgil (2004) find that BGs are characterized by a strong innovative capacity and a tendency to search for new markets, which leads them to become international at an earlier stage. This innovative capacity, in addition to generating new products and processes, should also facilitate the acquisition of new knowledge, which can then lead to a greater capacity to obtain improved firm performance. Therefore, the export performance of BGs is the result of their international focus, which refers to their knowledge of the international market and their international operations (Autio et al., 2000; Boso et al., 2013; Cavusgil and Knight, 2015). Such a focus often has a greater impact on performance when the firms simultaneously develop processes that incentivize innovation by providing greater capacity to their employees to compete in and understand international markets (Autio et al., 2000; Kraus et al., 2017; Zahra et al., 2000). Consequently, the following hypothesis is proposed:

Hypothesis 2 Innovative capacity positively moderates the relationship between an international orientation and export performance in BGs.

#### 2.2.2 International orientation and market dynamism

Besides the resource-based view that focuses on the internal factors that drive export performance, our study also considers institutional theory. We postulate that the influence of environmental factors on the BG's behavior is based on the influence of market dynamics and the favorability of the environment in the relationship between international orientation and export performance. The social, economic and political factors that frame society determine the survival of companies in the market and their decision-making processes (DiMaggio and Powell, 1983). Thus, market dynamism depends on a set of factors, results, and conditions at the firm level.

BGs can be confronted with greater problems in dynamic markets because such firms may not have yet developed response mechanisms to changes due to their recent creation and early internationalization. These problems may also stem from the fact that they are still at the stage of learning about the market (Rodriguez Serrano et al., 2012; Welter and Smallbone, 2011; Zhang et al., 2013). Given these circumstances, market dynamism tends to promote a higher degree of international orientation among BGs because of their need to frequently analyze their clients and competitors' actions and to develop organizational capacities that favor international activities. In line with Dimitratos and Plakoyiannaki (2003), BGs develop an international orientation that directs their attention to external market opportunities and the need to adapt to changing market conditions. Thus, BGs push strategic actions through their managers’ commitment to promoting various firm processes among employees and investing the resources that are necessary to initiate these processes and to generate a positive impact on export performance (Madsen, 1989). Therefore, the following hypothesis is proposed:

Hypothesis 3 Market dynamism positively moderates the relationship between an international orientation and export performances in BGs.

#### 2.2.3 International orientation and a favorable business environment

As noted previously, markets are subject to constant change and firms must evaluate their capacity to adapt to these changes. In international business environments, changes in global markets tend to be more dynamic because firms must adapt their processes to the regulatory framework of each country and the varying preferences of consumers (Shoham, 1999). In this context, one of the characteristics of the business environment that greatly affects firms’ struggle to adapt to international markets and remain in these markets is the favorability of the business environment, which is often considered to be an indicator of the stability and ability for growth for organizations in different international settings (Sutcliffe and Huber, 1998).

Previous research identified that favorable environments mean better access for firms to international markets. Firms often promote strategies that focus on obtaining better results because managers’ positive perceptions about their business environment can help them to acquire new commitments and to create better conditions within which to improve management practices in international activities (Elbanna, 2009; Elbanna and Alhwarai, 2012; Elbanna and Child, 2007). Thus, it is assumed that all types of firms, including BGs, promote a greater international orientation if they recognize a high likelihood of obtaining superior yields from export activities. Therefore, the following hypothesis is proposed:

Hypothesis 4 The favorability of the business environment positively moderates the relationship between an international orientation and export performance in BGs.

## 3 Methodology

### 3.1 Sample selection and data collection

Our study focuses on 112 exporters in major cities in Colombia (South America). The firms were classified based on three requirements for BGs defined by Escandón (2009) and Escandón and Hurtado (2012). The first criteria relates to a founding year of no more than seven years, that is, its year of creation was subsequent to 2007 (Jolly et al., 1992; Shrader et al., 2000; Zahra et al., 2000). Second, firms should be exporters and they should manage to achieve more than 25% of annual sales abroad or through consolidated international presence (Jantunen et al., 2008; Kuivalainen et al., 2007; Milanov and Fernhaber, 2009; Taylor and Jack, 2013). The third criteria, although in the case of Colombia does not show any results, relates to not include subsidiaries of foreign firms (Iborra et al., 1998; Knight et al., 2004; Madsen and Servais, 1997).

#### 3.1.1 Variable measurement

To collect the data, a questionnaire was developed that consisted of a set of general information questions on the firms, followed by the measurement scales that were taken from the literature. To measure international focus, we considered the scale that was developed by Sorensen and Madsen (2012), which was inspired by the multi-purpose indicator that was proposed by Knight and Cavusgil (2004) and Knight and Kim (2009), which has been used widely in other research. To measure export performance, we adapted the scale that was developed by Zou et al. (1998) to create a scale called EXPERF, which consists of three types of performance: Financial performance, strategic performance, and export satisfaction. To measure innovative capacity, we adapted a scale that was developed by Luo et al. (2005) that was based on the propositions of Hurley and Hult (1998) in which innovation is taken into account as an indispensable element for firm success. To measure the business environment variables, we used the Jaworski and Kohli (1993) scale to measure market dynamism, and we filtered five items that measured changes in the market that were due to changing client preferences or competitor behavior. For the measure of favorability of the business environment, we adapted the scale that was developed by Sutcliffe and Huber (1998) that indicates to what extent a business environment favors the stability and growth of firms. These scales were measured using a seven-point Likert scale (1 = completely disagree, 7 = completely agree). Details on the items in each scale are presented in Appendix 1.

Independent of the described variables, the other specific characteristics of a firm can also assert an impact on export performance. Therefore, we used firm size and international experience of the firm as control variables. We assumed that both variables positively affect export performance. Firm size was measured using the number of employees, and international experience was determined using the number of years that a firm has operated in international markets. For the regression analysis, we used a logarithmic scale to transform these variables and to correct asymmetries (Hair et al., 1999).

### 3.2 Measurement analysis

To analyze the psychometric properties of the scales that were used in this study, we conducted a correlation analysis and an exploratory factor analysis for each scale to identify its fit and unidimensionality. We then conducted a confirmatory factor analysis to assess the convergent and discriminate validity of the constructs. The factor analysis indicates that the data have a reasonable fit, and all of the measures show adequate reliability as measured by compound reliability indices (SCR) that exceed 0.6 (Bagozzi and Yi, 1988) and average variance extracted (AVE) measures that exceed 0.5 (Fornell and Larcker, 1981). The first indicator allows us to analyze the levels of reliability of all of the constructs that belong to a scale and level of contribution (Fornell and Larcker, 1981) and the AVE captures the ratio of the variance that is associated by a factor and the variance that is due to measurement error. We have concluded that the scales have the necessary reliability criteria (SCR and AVE). In addition, all of the loads correspond to their hypothetical factors, and the estimates are significant given their high t-values, thus providing evidence of convergent validity (Bagozzi and Yi, 1988). Discriminant validity is confirmed in all of the scales by verifying that the value “1” is not present in the confidence intervals that were calculated between each of the construct pairs (Anderson and Gerbing, 1988). We also confirmed discriminate validity because we obtained in each scale an average variance that was extracted by the underlying construct that is greater than the variance shared with another latent construct (Hernandez-Espallardo et al., 2010). The resulting fit statistics are as follows: χ^2^(485) = 799.82; GFI = 0.88; RMSEA = 0.041; SRMR = 0.047; CFI = 0.95; TLI (NNFI) = 0.95. Table 1 presents the descriptive statistics, the correlation coefficients, and the compound and average reliability of the variance that was extracted for each measurement scale.

**Table 1:** Descriptive statistics, correlation matrix, and compound and average reliability of the variance extracted.

		Mean	SD	1	2	3	4	5	SCR	AVE
1	International orientation (IO)	4.39	0.31	0.51					0.80	0.51
2	Export performance (EP)	5.25	0.05	0.40	0.70				0.92	0.70
3	Innovative capacity (IC)	5.14	0.13	0.42	0.27	0.5			0.75	0.51
4	Market dynamism (MD)	4.28	0.54	0.35	0.19	0.24	0.51		0.76	0.51
5	Favorability of business environment (FE)	4.39	0.43	0.24	0.30	0.38	0.22	0.6	0.83	0.61
6	Size of firm	31.2	41.1	0.19	0.68	0.57	0.41	0.51		
7	Experience of firm	4.65	0.04	0.35	0.51	0.61	0.69	0.38		

## 4 Results

To rule out the presence of multicollinearity in the indicators that make up each construct value's factor inflation variance (VIF), condition indices that are well below the threshold of 10 and not as close to 0 are used (greater than 0.1); they indicated no multicollinearity problems (Hair et al., 1999). Additionally, the bi-variate correlations with values well below the critical value for the presence of multicollinearity (0.8) were obtained, allowing us to infer that they measured different concepts.

A Harman factor was used through the potential barrier of common method variance, which is a frequent problem when all of the variables are measured with the same instrument (questionnaire), which can potentially lead to the variance being attributed to the method and not the constructs (Podsakoff et al., 2003). Therefore, a factor analysis was developed and confirmed that the eigenvalues of all of the factors were greater than one and represent more than 68% of the total variance.

Moreover, it is important to note that the survey respondents expressed interest in the study and its subsequent results, and therefore their responses may prevent response bias. Additionally, a high response rate was achieved due to the development of surveys and forecasts some respondents found to be personally unacceptable or they were unable to answer or they simply were not available to respond. In this sense, a subset of the companies that met the conditions of being born global was selected.

However, although we made a great effort to avoid problems of bias, it is clear that these challenges are inherent in studies of this nature, but they do not invalidate the results.

**Fig. 2:**
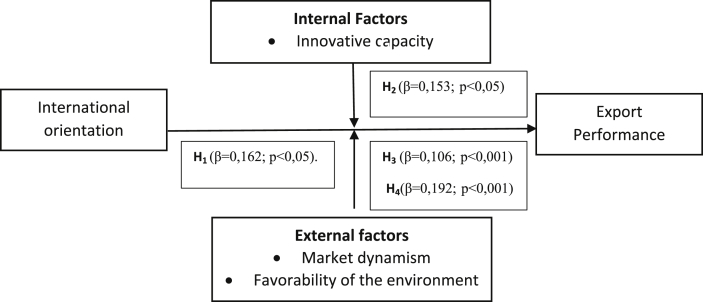
Model.

The model estimation was performed using a hierarchical linear regression (see Fig. 2). Before estimating the model, the independent variables were mean centered to reduce the multicollinearity that results from the presence of the multiplication terms that are used to estimate the moderating effects (Aiken and West, 1991). We started by estimating the model with the control variables, and subsequently, we estimated a model including all of main effects. Next, we estimated a second model, including the moderating effects. This form of model estimation complies with the parsimony principle that establishes the introduction of higher-order terms only if they significantly improve a model's explanatory capacity (Aiken and West, 1991), i.e., the estimated main effects model explained a significant amount of variance (F = 30,20; p < 0.001; R^2^ = 0,28; R^2^ adjusted = 0,28). When we included the moderating effects, the R^2^ value increased by 8,9% (p < 0.001). The results of the final model that includes the main effects and the moderating effects are presented in Table 2.

**Table 2:** Results of the estimated model.

Variable	Coefficient	Control Model		Main Effects Model		Theoretical Model	
		Standardized Coefficient	P-Value	Standardized Coefficient	P-Value	Standardized Coefficient	P-Value
Constant	b_0_	2.57	0,000	2.20	0,000	2.29	0,000
Size of firm	b_1_	0,15	0,000	0,078	0,048	0,369	0,000
Experience of firm	b_2_	0,48	0,000	0,399	0,000	0,079	0,043
IO	b_3_			0,086	0,180	0,234	0,451
IC	b_4_			0,136	0,003	0,142	0,002
MD	b_5_			0,229	0,000	0,239	0,000
FE	b_6_			-0,017	0,765	-0,017	0,765
IO*IC	b_7_					0,070	0,058
IO*MD	b_8_					0,116	0,041
IO*FE	b_9_					-0,182	0,000

R^2^ (R^2^ Adjusted)		0,28 (0,28)		0,369 (0,357)		0,41 (0,391)	
F value (F-probability)		62.75 (0,000)		30,20 (0,000)		23,44 (0,000)	
R^2^ Changes (R^2^ adjusted)		-		0,089 (0,357)		0,041 (0,391)	

Hypothesis 1 proposes that an international orientation has a positive effect on the export performance of BGs, and the results support this prediction (β = 0.62; p < 0.05). Hypothesis 2 proposes that an innovative capacity positively influences the effects of an international orientation on export performance, and Table 2 shows a significant, positive interaction effect in line with this hypothesis (β = 0.153; p < 0,05). Hypothesis 3 proposes that market dynamism positively influences the effects of an international orientation on export performance, and a significant, positive effect is obtained (β = -0,106; p < 0,001). Finally, according to Hypothesis 4, a favorable business environment should positively influence the effects of an international orientation on export performance, and the results support this prediction because a significant, positive interaction coefficient is obtained (β = 0,192; p < 0.05).

To interpret the results of the main effects and the moderating effects, we followed the procedures that were proposed by Aiken and West (1991) and graphically represented the interactions for which the high- and low-level values of the variables included in the interaction were estimated. For the low values, we defined a standard deviation that is lower than the mean, and for the high values, we defined a standard deviation above the mean. To estimate the results, the effects of the other variables were maintained constant.

**Fig. 3:**
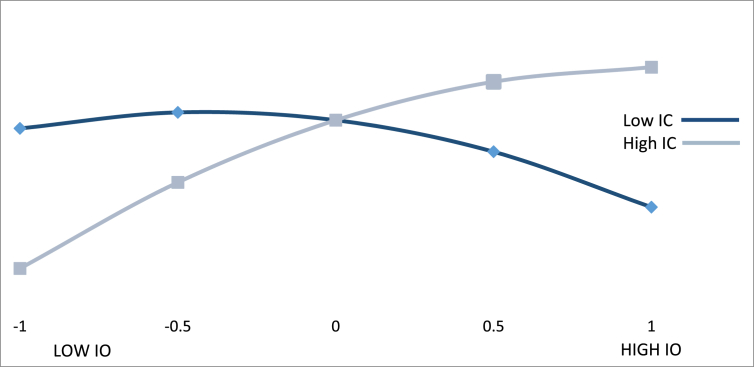
Moderating effect of international orientation *x* innovative capacity.

**Fig. 4:**
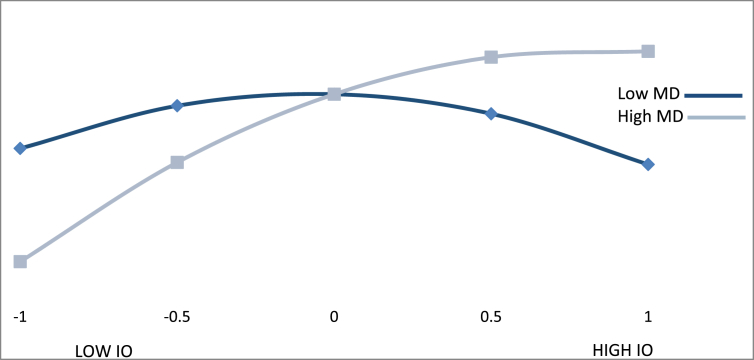
Moderating effect of international orientation *x* market dynamism.

**Fig. 5:**
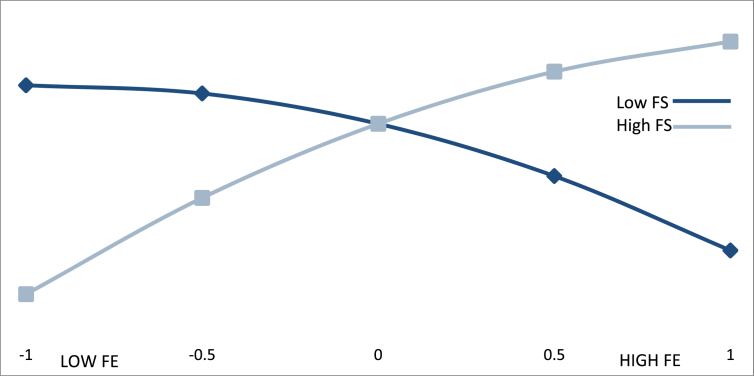
Moderating effect an international orientation *x* favorable business environment.

Figs. 3, 4, and 5 show the effect of an international orientation on export performance for different values of the moderating variables of innovative capacity, market dynamism, and favorability of business environment. The Y-axis of each graph represents the values that were obtained for export performance (EP) when different values of innovative capacity (IC) (Fig. 3), market dynamism (MD) (Fig. 4), and favorability of business environment (FE) (Fig. 5) are introduced into the estimated regression function. On each X-axis, we present the different values of an international orientation (IO).

Fig. 3 shows the moderating effect of innovative capacity, which demonstrates that the positive impact of an international orientation on export performance is subject to high innovative capacity in a firm. At low IO levels (-1.4), when IC also has low levels (-1.039), the effect of IO on EP is 0.622 with a standard error of 0.747 and a t-value of 0.863 (P > 0.1). For high values of IC (+1.039), the effect increases to 6.174 with a standard error of 1.26 and a t-value of 4.9 (P < 0.01). For high levels of IO (+1.4), when IC has a low value (-1 standard deviation, -1.039), the effect of IO on EP is -2.276 with a standard error of 1.415 and a t-value of 2.916 (P < 0.01). This effect increases to 1.426 (t = 1.769; P < 0.10) when IC has high levels.

The results show that IO is more effective on EP when it is accompanied by high levels of IC. Thus, the variables seem to complement each other, as postulated in Hypothesis 2. Nevertheless, we needed to prove that the moderating effect of IC is significant. To do so, using prior values, we obtain a jumping effect of IO on EP that equals 5.554 (t = 5.554; P < 0.01) when we went from low IC values to high IC values. Thus, we can conclude that IO is more effective in the presence of high levels of innovative capacity. Innovative firms will therefore have a greater chance of obtaining better results. When the managers of BGs have an international orientation but innovative capacity is not a feature of their organization, the firms are less likely to achieve good export performance.

Fig. 4 shows that the positive implications of an international orientation on export performance are subject to the high market dynamism of BGs. For low values of IO (-1 standard deviation, -1.4), the change of the IO effect on EP in low values of MD (-1 standard deviation, -0.862) is 1.826 with a standard error of 0.49 and a t-value of 3.727 (P < 0.01). For high values of MD (+0.862), the effect is 4.97 with a standard error of 0.924 and a t-value of 5.38 (P < 0.01).

Moreover, for high values of IO (+1.4) and a low MD (-0.862), the effect of IO on EP is -2.924 with a standard error of 1.23 and a t-value of -2.38 (P < 0.05). For high values of MD (+0.862), the effect of IO on EP is 0.22 with a standard error of 0.944 and a t-value of -0.23 (P > 0.1). In all of the IO levels are considered, we obtain a difference in the effect of IF on EP between the low levels and high levels of MD. This difference is 3.14, and t = 3.309 (P < 0.01). This result allows us to confirm Hypothesis 4, i.e., market dynamism moderates the relationship between IO and EP. This moderation is present for low values of MD and both high and low values of IO. However, for small MD values with high IO values, the relationship with export performance is improved, but when the values of IO are high, a saturation process begins that affects the relationship with export performance in a significant but negative way. However, although the moderating effect of market dynamism is not significant at high levels of MD and high levels of IO, the change in the effect when passing from low levels to high levels of MD is significant and allows us to confirm that there is a moderating effect of market dynamism on the relationship between IO and export performance.

Finally, according to Fig. 5, the more favorable the business environment in which BGs operate, the greater the positive influence of an international orientation on export performance. For low IO values (-1 standard deviation, -1.4), the change of the effect of IO on EP in low values of FE (-1 standard deviation, -0.957) is 1.945 with a standard error of 0.58 and a t-value of 3.353 (P < 0.01). For high values of FE (+0.957), the effect is 5.86 with a standard error of 0.946 and a t-value of 6.194 (P < 0.01).

Moreover, for high values of IO (+1.4) and low values of FE (-0.957), the effect of IO on EP is -3.045 with a standard error of 1.46 and a t-value of -2.08 (P < 0.05). For high values of FS (+0.957), the effect of IO on EP is 0.55 with a standard error of 0.996 and a t-value of -0.55 (P > 0.1). At all of the IO levels considered, we obtain a difference in the effect of IO on EP between low levels and high levels of FS. This difference is 4.15, t = 3.819 (P < 0.01). This result allows us to confirm Hypothesis 4, i.e., a favorable business environment moderates the relationship between IO and EP. This moderation is present with low values of FE and both high and low values of IO. However, for low FE values with high values of IO, the relationship with export performance is improved, but if the values of IO are high, a saturation process is generated that affects the relationship with export performance in a significant but negative way. Moreover, although the moderating effect of a favorable business environment is not significant at high levels of FE and high levels of IO, the fact that the change in the effect from low levels of FE to high levels is significant allows us to confirm that there is a moderating effect of a favorable business environment on the relationship between IO and export performance.

In summary, this study contributes to the literature because it enriches our knowledge of BGs and export performance and their determining factors. Additionally, the study allows us to shed light on other research that assumes that an international orientation is a strategy (Jantunen et al., 2008; Knight and Kim, 2009). It furthermore allows us to examine the various variables that affect the impact of international orientation on export performance by demonstrating that there are both internal and external factors that influence this relationship (Hagen and Zucchella, 2014; Madsen and Servais, 1997; Rialp et al., 2005).

## 5 Discussion

The purpose of our study consisted in exploring empirically how BGs achieve superior international performance from a capability perspective. Particularly we were interested in the relationship between the BGs international orientation and its export performance mediated, on the one hand, by its innovation capacity (internal factor), and, on the other hand, by the market dynamism and the favorability of the business environment (external factors).

Through the conceptual model we have chosen and the empirical context of our research, we make the following contributions to research on the phenomenon of BGs. Firstly, our research is the first to use the construct of international orientation in the context of BGs. We therefore provide important explanations of how BGs are able to achieve superior export performance through particular capability antecedents (Knight and Liesch, 2016). Secondly, we contribute to a better understanding of the effects of variables related to the environment and context of BGs in order to achieve superior export performance (Knight and Liesch, 2016). Thirdly, we focus on BGs from Colombia and therefore contribute to research from a developing country perspective (Baier-Fuentes et al., 2019; Oyna and Alon, 2018).

In the following we present a summary of the results, followed by a discussion of the theoretical contributions, and conclude with the managerial implications of our findings.

### 5.1 Summary of results

We predicted a positive relationship between the BGs international orientation and its export performance and the result supports this prediction. The results further show that international orientation has a greater effect on export performance through the internal and external mediator variables introduced into the model. Related to the innovative capacity of the BG as an internal firm capability, our results indicate that international orientation is more effective on export performance when it is accompanied by high levels of innovative capacity. Related to the market dynamism as an external market factor, our results indicate that the positive relationship of the international orientation on export performance are subject to the high market dynamism where BGs operate in. Related to the favorability of the business environment as an external factor, our results show that the more favorable the business environment in which BGs operate, the greater the positive influence of an international orientation on export performance. Hence, the results confirm the moderating effect of all internal and external variables on the relationship between international orientation and export performance.

### 5.2 Theoretical contributions

Our results confirm the findings of literature that establish a strong and positive relationship between the firm's capabilities and its export performance (Jantunen et al., 2008; Knight and Cavusgil, 2004; Knight and Kim, 2009; Sorensen and Madsen, 2012). To the best of our knowledge, our study is the first to apply the construct of an international orientation (Knight and Kim, 2009) to the context of BGs. We therefore provide explanations of how BGs are able to achieve precocious internationalization and superior export performance based on the mindset of their managers towards international markets (Knight and Liesch, 2016). Or as Sorensen and Madsen (2012, p. 426) puts it: “International orientation is a question of mindset (whether managers see the world as their market place as well as their motivation to deal with international customers and partners), but it is also critical that top management has a clear commitment of resources and develops an organizational culture that motivates employees' behavior in the direction of international activities. […] [T]he mindset of the managers should reflect the international strategies of the firm, implying that top management encourages employees to actively explore possibilities in foreign markets, and stresses that organizational agility and adaptability is crucial to compete successfully in foreign markets.”

The international orientation of the BG therefore constitutes an important intangible resource that contributes to the sustained competitiveness of the firm (Barney, 1991). Furthermore, we show that international orientation is indeed an important internal capability for the BG able to offset the lack of tangible resources in order for the smaller firm to achieve early and rapid internationalization (Autio et al., 2000; Cavusgil and Knight, 2015; Zahra et al., 2000). In their recent review of the literature on BGs, Paul and Rosado-Serrano (2019), consider the international orientation of the entrepreneur as one of the important characteristics that differentiate gradual internationalizing firms from BGs. Therefore, it is therefore surprising that no previous empirical research looked at the international orientation-performance relationship in the context of BGs.

Besides, establishing the international orientation as an important internal capability able to contribute to superior export performance of the BG, we provide insights into its moderating effects. Moderating effects are important as both internal and external drivers are found to impact the early and accelerated internationalization of new ventures (Hagen and Zucchella, 2014; Madsen and Servais, 1997; Mathews and Zander, 2007; Rialp et al., 2005).

In line with previous export literature (Pla-Barber and Alegre, 2007; Filatotchev et al., 2009) and BGs (Cavusgil and Knight, 2015; Knight and Cavusgil, 2004), our results confirm the importance of innovative capacities for early internationalization. Particularly, our results indicate that international orientation is more effective in the presence of high levels of innovative capacity. However, our results also show an effect of diminishing return of export performance for high levels of innovative capacity once a certain level of international orientation is achieved (see Fig. 3). In other words, beyond a certain point, the additional exploration of new business opportunities for export markets, the additional development and adaptation of products for exports, and the additional promotion of export activities among employees, does not add as much value as their associated costs for investing in an innovative capacity.

The effect of a diminishing return is most evident for high levels of international orientation at high levels of market dynamism (see Fig. 4). This means that beyond a certain point and at high levels of market dynamism, it does not justify to dedicate additional resources to increase the firm's international orientation as it does not contribute to an increase in export performance. This effect is less pronounced for operating in favourable business environments (See Fig. 5). In favourable business environments, an additional increase in the BG's international orientation still leads to a significant increase in its export performance.

The differences in these diminishing effects is best explained by the nature of markets where BGs are operating in. Market growth is generally considered to have a positive impact on the firm's international performance (Whitelock, 2002). A favourable business environment facilitates the BG to establish its market niche and to become a dominant player in a less competitive environment. Research found that BGs are able to benefit from their niche's growth potential especially in the short-term (Efrat and Shoham, 2012). Therefore, BGs operating in favourable, growth markets, are still able to experience increased export performance while increasing their committment towards these international markets. BGs are also characterized for being active players in turbulent markets where they are able to exploit technological trends for creating their niche offer (Knight and Cavusgil, 2004). Frequent changes, however, require a constant adaptation to new trends. The BGs in our sample seem to reach a saturation point regarding their capability to continuously adapt to market trends, so that, an increase in their international orientation does not lead to a significant increase in export performance at high level of market dynamism. These diminishing effects, to a lesser extend for innovative capacity and favourable business environments and to a greater extend for market dynamism, have important managerial implications as we will explain below.

Our current understanding on BGs focuses largely on the description of their formation and to a lesser extent on the determinants of their performance (Gerschewski et al., 2015; Rialp et al., 2005). Our research provides much needed insights into the determinants of international performance of BGs (Knight and Liesch, 2016; Oyna & Alon, 2018; Paul and Rosado-Serrano, 2019). Our results emphasize the importance of an international orientation as a decisive capability for the BG in order to achieve a sustained competitive advantage. This resembles the general view of the importance of intangible resources for the likelihood of early internationalization. However, the possession of a strategic international orientation alone does not explain the superior export performance of our BGs. Both internal and external drivers impact on this relationship and are able to enhance its effects: 1) The innovative capacity is an important characteristic that defines a BG (Cavusgil and Knight, 2015; Knight and Cavusgil, 2004) and our results indicate that it also enhances the export performance of the BG; 2) BGs are known to operate in turbulent markets and high-growth environments (Efrat and Shoham, 2012; Knight and Cavusgil, 2004). Our results show that BGs are not only active in these markets but that their operations in these markets also contribute to an increased performance. Hence, we also contribute to extant research by analyzing the effects of variables related to the environment and context of BGs (Knight and Liesch, 2016).

The country context of our BGs also contributes to a better understanding of this type of firm from a developing country perspective (i.e. Colombia). As noted by many (Baier-Fuentes et al., 2019; Oyna and Alon, 2018), we lack an understanding about the characteristics and performance drivers of BGs from Latin America. According on Barbosa and Ayala (2017) Born global had been studied in Latinoamerican countries, especially in Brazil, Colombia and Chile, but it tried to analyze and contributed in Born global definition. Other hand, it researcher had emphasis in tech sector and qualitative methods to analyze internationalization behavior, role of networks and speed of internationalization (De Mello et al., 2019).

### 5.3 Managerial implications

Developing a global vision, where the firm sees the world, not just Colombia, as its market, is most probably the most important determinant for export success of Colombian BGs. Founders and managers of these firms are advised to develop a global outlook for their operations and to promote vigorously the exploration of new business opportunities on export markets. Policy makers can contribute to the development of an international orientation by promoting exports and facilitate international trade fair participation and export missions to potential firms. The development of an international orientation as a firm capability should most probably be one of the most important development objectives for export promotion agencies in Colombia as it helps to offset the resource scarcity of these small and medium-sized firms in order to make these firms more competitive in export markets.

However, the sole commitment towards export markets is not sufficient. The firm also needs to develop an innovative capacity and target appropiate markets. Compared to the external factors of the environment, the development of a capability for innovation is more challenging for the BG. It is similar to the international orientation, an intangible resource that needs to be developed over time and requires constant attention in order to be maintained as a capability. The environmental drivers are easier to control. Nevertheless, spotting attractive international markets requires significant and constant attention on behalf of the managers of BGs. Policy makers can assist firms by providing necessary market intelligence in order to survey international markets for their trends and growth potential.

Due to the diminishing effects on export performance we observed in our results, founders and managers of Colombian BGs should be aware that the development of an international orientation reaches a saturation point. Beyond that point, the contribution of additional international orientation does not contribute significantly to more export performance. Hence, the development of an international orientation reaches a tipping point and no further investments for strengthening the international orientation are necessary.

Our study, like all empirical quantitative research, is subject to limitations. We are recognizing that our quantitative method have two main limitations. First, hierarquical model is a transverse method and does not include a dynamic behavior for our variables and this information might be associated a punctual economic effect. Other hand, this method has a high tendency to increase our results, especially R2 adjust, due to it has more variables.

Future research should consider the following aspects building on the findings of our study. First, we researched capabilities and external drivers applying a static perspective. The survey instrument we used allowed us to construct a one-shot image of the current relationship between international orientation ad export performance. However, we were not able to provide insights into the dynamics of capabilities. Capabilities are subject to change and development (Al-Aali and Teece, 2014). Future research should consider the changing and dynamic nature of capabilities by capturing their differences over time. This also applies for the evolving nature of the markets and environments the BGs operate in.

Second, the focus of our research has been on a particular capability – the international orientation of the BG. Other capabilities such as a learning orientation, market orientation, international entrepreneurial orientation, and international growth orientation have shown to be positively related to the international performance of BGs (Gerschewski et al., 2015). These capabilities should be taken into consideration along international orientation for future research on BGs as possible intangible resources allowing a sustained competitive advantage.

Third, our sample consists of BGs from Colombia. In Latin America, Colombian and Chilean entrepreneurs particularly show a high international orientation (Amoros et al., 2015). Therefore, comparing the results of the Colombia study with other countries would be useful in order to derive more generalizable findings.

Finally, the institutional environment is important to keep into consideration for the study of BGs (Szyliowicz and Galvin, 2010). The impact of institutional forces on entrepreneurship is particularly heightened in emerging economies (Gupta et al., 2014; Kiss et al., 2012; Peng et al., 2008) and different formal and informal institutional environments seem to impact the performance of BGs in Latin America (Alvarez et al., 2014; Torkkeli and Fuerst, 2018). Future studies should therefore keep in mind the impact of the institutional environment on the export performance of BGs.

## Declarations

### Author contribution statement

D. Escandon-Barbosa, J. Rialp-Criado, S. Fuerst, G. Castro-Aristizabal: Conceived and designed the analysis; Analyzed and interpreted the data; Wrote the paper.

A. Rodriguez-Orejuela: Contributed analysis tools or data.

### Funding statement

This research did not receive any specific grant from funding agencies in the public, commercial, or not-for-profit sectors.

### Competing interest statement

The authors declare no conflict of interest.

### Additional information

No additional information is available for this paper.

## Figures and Tables

**Table:** 

Items	Standardized Loading	t value	Reliability SCR^a^ AVE^b^
***International Orientation (adapted from***Sorensen and Madsen, 2012***)*** The desire for growth is a strong motive for the expansion of international activities The possibility of increased profits is a strong motive for international expansion We see the world, not just Colombia, as our firm's market We find it better to expand our export activities cautious and gradually Active exploration of new business opportunities on export markets Strong capability to develop and adapt new and existing products/services Our priority is success with export activities Development of human and other resources that may contribute to successful export	0.64 0.68 0.75 0.77 0.71 0.70 0.69 0.70	11.95 12.93 14.57 15.25 14.30 14.26 14.20 14.26	SCR = 0.80 AVE = 0.51
***Export Performance (adapted from***Zou et al., 1998***)*** has been very profitable has generated a high volume of sales has achieved rapid growth has improved our global competitiveness has strengthened our strategic position has significantly increased our global market has been very satisfactory has had been very successful has exceeded our expectations	0.74 0.73 0.90 0.87 0.77 0.81 0.83 0.82 0.79	14.93 14.63 20.15 18.92 15.25	SCR = 0.89 AVE = 0.66
***Innovative Capacity (IC) (adapted from***Luo et al., 2005***)*** Technical innovations based on research, are easily accepted The company leads actively seeks new ideas The staff of the company easily accepts innovation company The staff is rewarded for new ideas, even if they do not work Innovation is perceived as an opportunity, not as a risk	0.72 0.76 0.78 0.75 0.82	14.24 15.40 15.95 15.48 17.18	SCR = 0.88 AVE = 0.59
***Market dynamism (MD) (adapted from***Jaworski and Kohli, 1993***)*** In our kind of business, costumers' product preferences change quite a bit over time Our customers tend to look for new products all the time Our customers are very price-sensitive, but on other occasions, price is unimportant Demand for our products and services from customers who never bought them before New customers tend to have product-related needs that are different of our customers We cater to many of the same customers that we used to in the past	0.60 0.85 0.68 0.75 0.69	10.35 14.67 11.88 12.15 12.18	SCR = 0.76 AVE = 0.51
***Favorability of Business Environment (FS) (adapted from***Sutcliffe and Huber, 1998***)*** The demand in international markets is growing and will continue to grow Investment or marketing opportunities for firms are very favorable at the present time There are great opportunities for businesses to expand their international markets There are available resources for further growth in these markets	0.76 0.74 0.85 0.72	14.55 14.06 16.84 15.22	SCR = 0.83 AVE = 0.61

Adjustment stadistics with 5 constructs:: χ2(485) = 799.82; GFI = 0.88; RMSEA = 0.041; SRMR = 0.047; CFI = 0.95; TLI (NNFI) = 0.95. a Scale composite reliability (ρc=(∑λi)2 var (ξ)/[(∑λi)2 var (ξ) + ∑θii]; (Bagozzi and Yi, 1998)).^b^ Average variance extracted (ρc=(∑λi2 var (ξ))/[∑λi2 var (ξ) + ∑θii]; (Fornell and Larcker, 1981). c anchors: 0 = Totally disagree; 7 = Totally agree. (*) Item eliminated during the validation process of the scale.
